# Bilateral thalamic stroke due to occlusion of the artery of Percheron: A case report and literature review

**DOI:** 10.1002/ccr3.8155

**Published:** 2023-11-08

**Authors:** Eliz Achhami, Seshkanta Lamichhane, Satyam Mahaju, Lukash Adhikari, Ashim Kandel, Anubhav Poudel

**Affiliations:** ^1^ Sukraraj Tropical & Infectious Disease Hospital Kathmandu Nepal; ^2^ Bir Hospital Kathmandu Nepal; ^3^ Patan Academy of Health Sciences Lalitpur Nepal

**Keywords:** artery of Percheron, bilateral thalamic infarction, clinical impact, midbrain function, neurovascular complexity

## Abstract

Bilateral thalamic infarction resulting from the occlusion of the artery of Percheron (AOP) is a rare cerebrovascular event with distinctive clinical presentations. This case report explores the intricate relationship between vascular anatomy, midbrain function, and clinical manifestations. A 48‐year‐old male farmer with a history of diabetes mellitus presented with sudden‐onset visual disturbances, diplopia, bilateral eyelid drooping, and loss of consciousness. Extensive evaluations, including advanced imaging techniques, led to the diagnosis of bilateral upper midbrain infarction involving AOP. This case underscores the complexity of neurovascular interactions, highlighting the importance of precise diagnosis, and tailored management in addressing rare cerebrovascular conditions.

## 1 BACKGROUND

**FIGURE 1:**
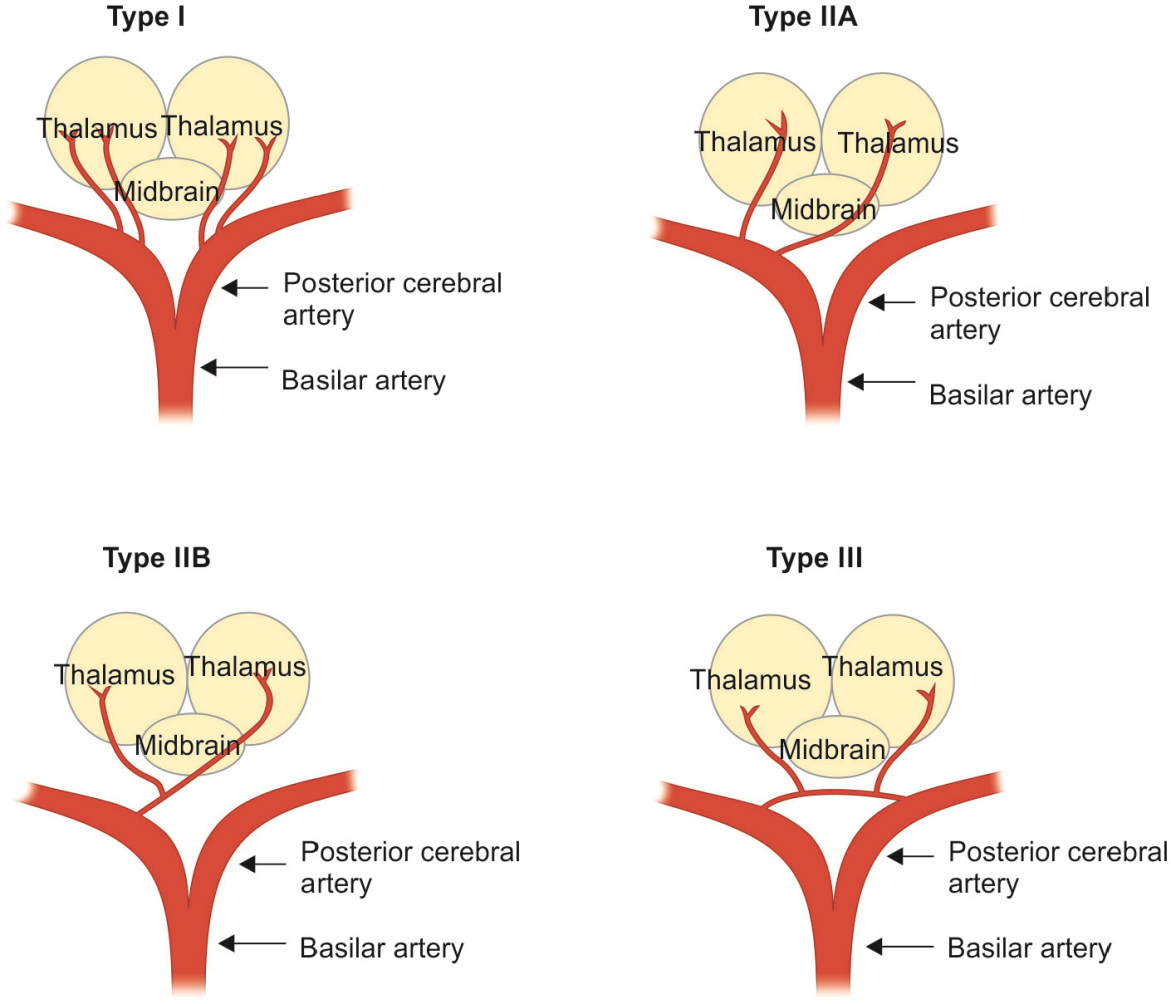
A diagram illustrating the four variants of the arterial supply to the thalamus.

The blood supply to the thalami and midbrain comes from both the anterior (internal carotid arteries) and posterior (vertebro‐basilar system) circulations, and various variations in this supply have been reported.^1^, ^2^ Typically, the anterior circulation provides blood to the anteroinferior region of the thalami and midbrain through thalamoperforator arteries that arise from the posterior communicating arteries (Pcom). On the other hand, the posterior circulation supplies the medial aspects of the thalami and midbrain via branches originating from P1 segments and the lateral and superior aspects via branches arising from P2 segments of the posterior cerebral arteries (PCAs).^3^ Coma caused by bilateral paramedian thalamic infarction resulting from the occlusion of the artery of Percheron (AOP) is an uncommon vascular condition, and its exact prevalence is not yet determined. Gérard Percheron first described the AOP in 1973 as an anatomical variant of the paramedian arteries originating from segment P1 of the PCA.^1^ The suggested mechanism involves the occlusion of a single central thalamic perforating artery, which was previously described by Percheron^1^ (Figure 1). The AOP variant is found in around 4%–12% of the population.^4^ In two large stroke series studies, characteristic AOP infarct patterns accounted for 0.1%–2% of all ischemic strokes, highlighting that this type of ischemic stroke is exceptionally rare.^5^

In this case report, we present the clinical profile of a 48‐year‐old male farmer with a known history of diabetes mellitus who presented with sudden‐onset blurry vision, diplopia, bilateral drooping of eyelids, and loss of consciousness. The patient's initial management involved treatment for suspected meningioencephalitis, given the presence of fever and unresponsiveness. However, further investigations, including brain MRI and MRA, led to the diagnosis of bilateral upper midbrain infarction involving the AOP.

## 2 CASE PRESENTATION

A 48‐year‐old male, a farmer by profession, with a known history of diabetes mellitus for 5 years and on metformin 500 mg twice a day, presented with a sudden onset of blurry vision, diplopia (double vision), and bilateral drooping of eyelids and loss of consciousness while he was working in the field. He was immediately taken to the local hospital where he had a fever of 101°F. At the local hospital, he received intravenous fluids, intravenous paracetamol, and intravenous ceftriaxone. Due to the severity of his condition, he was subsequently referred to a tertiary care center for further management.

Upon arrival at the tertiary care center, the patient had a Glasgow Coma Scale (GCS) score of E2V3M4, indicating a severe neurological impairment. His blood pressure was recorded at 130/70 mmHg and his pulse was elevated at 110 beats per minute. The respiratory rate was 20 breaths per minute, and his temperature had decreased to 99°F. While his oxygen saturation was 95% in room air, his bilateral eyes exhibited lateral deviation, and the pupils were nonreactive, measuring 5 mm in size. However, he did maintain intact cough and gag reflexes, and neck rigidity was notably absent. The patient responded with limb movements upon experiencing painful stimuli. During the cardiovascular, respiratory, and abdominal examinations, no abnormalities were note.

The patient underwent a non‐contrast CT scan of the head, which did not show any significant findings. However, due to the presence of fever and unresponsiveness, the medical team decided to initiate treatment for meningioencephalitis. Intravenous ceftriaxone, intravenous vancomycin, intravenous acyclovir, and intravenous dexamethasone were administered in line with this suspected diagnosis. Subsequently, a lumbar puncture was performed, and cerebrospinal fluid (CSF) analysis revealed a total cell count of 6 with 100% lymphocytes, glucose level of 88 mg/dL, protein level of 42 mg/dL, and an ADA (adenosine deaminase) level of 7.5. Despite extensive testing, including CSF gram stain, culture sensitivity, and gene xpert analysis, no specific causative agent was identified. PCR testing for herpes simplex 1 and 2 in the CSF also returned negative results. The patient's laboratory findings at the time of admission and at the time of discharge are summarized in Table 1.

**TABLE 1:** Summary of his blood investigation during his hospital stay.

Parameter	Value	Reference range
TLC (total leukocyte count)	10,400	4000–11,000/μL
Neutrophil	83	40%–75%
Lymphocytes	13	20%–40%
Monocytes	3	2%–8%
Eosinophils	2	0%–6%
Platelets	245,000	150,000–450,000/μL
Hemoglobin	13	12–16 g/dL
PT/INR	13/1	0.8–1.2
Urea (ur)	90	20–40 mg/dL
Creatinine (cr)	0.7	0.6–1.2 mg/dL
Sodium (Na)	146	135–145 mmol/L
Potassium (K)	4	3.5–5.1 mmol/L
Total bilirubin (TB)	1	0.2–1.2 mg/dL
Direct bilirubin (DB)	0.3	0–0.3 mg/dL
AST (aspartate aminotransferase)	20	10–40 U/L
ALT (alanine aminotransferase)	28	7–56 U/L
ALP (alkaline phosphatase)	59	38–126 U/L
Total protein	5.4	6.0–8.3 g/dL
Albumin	2.7	3.4–5.4 g/dL
Free T3 (fT3)	4.37	2.3–4.2 pg/mL
Free T4 (fT4)	14	9–23 pmol/L
TSH (thyroid stimulating hormone)	1.12	0.4–4.0 μIU/mL
Calcium (Ca)	8.9	8.5–10.5 mg/dL
Magnesium	2.04	1.7–2.3 mg/dL
VDRL (venereal disease research laboratory)	Non reactive	Non reactive
Hepatitis B	Non reactive	Non reactive
Hepatitis C	Non reactive	Non reactive
HIV	Non reactive	Non reactive
HbA1c	7	4.0%–5.6%
Dengue serology	Negative	Negative
Japanese encephalitis serology	Negative	Negative
HSV1/2 PCR	Negative	Negative

**FIGURE 2:**
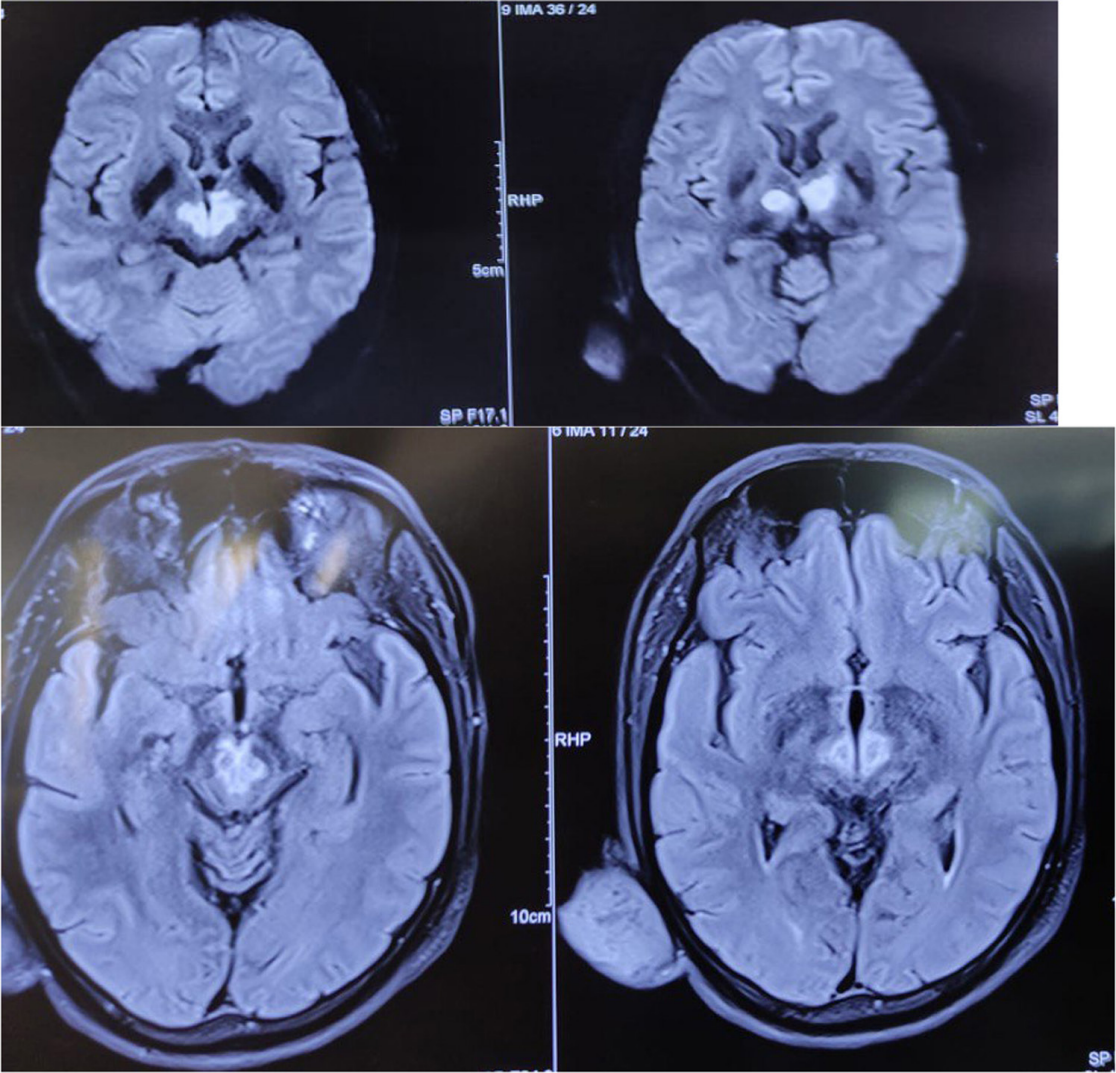
MRI of the brain revealing T2/FLAIR signal intensity lesions in bilateral thalamus, including the midbrain.

**FIGURE 3:**
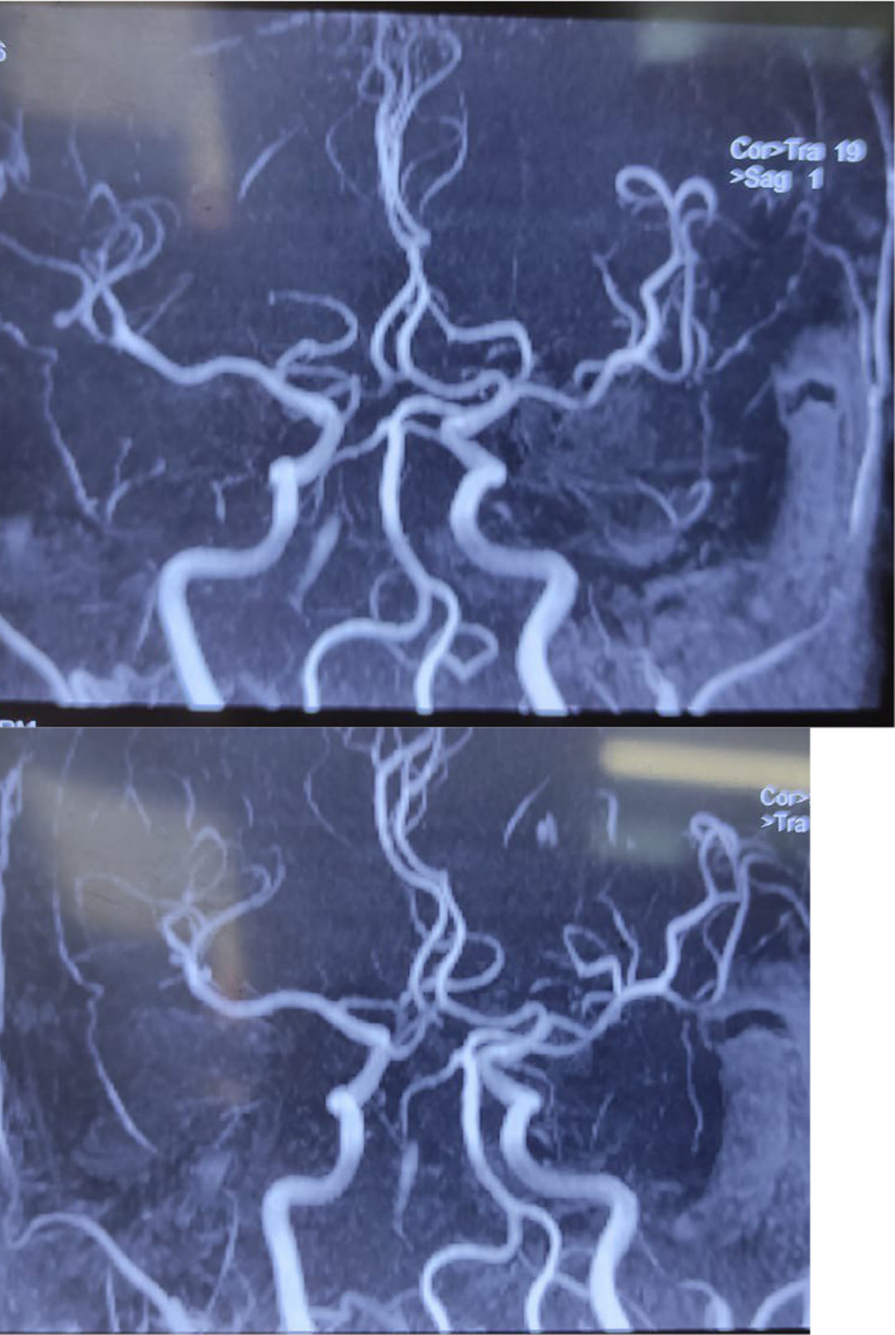
MRA demonstrating non‐visualization of the right P1 segment, and P1 segment arising from the right posterior communicating artery.

An MRI of the brain was conducted, revealing altered T2/FLAIR signal intensity lesions in bilateral thalamus, including the midbrain, with restricted diffusion, and blooming artifact (Figure 2). Further assessment through MRA demonstrated non‐visualization of the right P1 segment, and it was noted that the P1 segment was arising from the right posterior communicating artery (Pcom) (Figure 3). To investigate further, serology for dengue and Japanese encephalitis was performed, but the results came back negative. Considering the MRI findings, there was a suspicion of bilateral upper midbrain infarction involving the AOP.

Because of more than 24 h of documented stroke diagnosis as he was known at his baseline, the patient was not a candidate for tissue plasminogen activating factor (tPA). The neurosurgery team recommended conservative management. The patient continued to improve. Within the first 48 h, the patient was confused but awake and responsive to minimal commands. In light of the patient's condition remaining stable with a GCS of E3V4M4 over 3 weeks of treatment, a decision was made to discharge the patient for home care and management. Medications such as tab aspirin 75 mg and tab rosuvastatin 20 mg were prescribed, likely to manage the stroke‐related aspects of the condition. After 1 month of follow‐up, the patient continues to experience difficulty in opening his eyes spontaneously. However, they are showing positive signs of responsiveness by communicating verbally and demonstrating spontaneous movement in all their limbs.

## 3 DISCUSSION AND CONCLUSIONS

Bilateral thalamic infarction due to AOP occlusion is an uncommon cerebrovascular event with distinctive clinical manifestations. In this case, the patient, a 48‐year‐old male farmer with a history of diabetes mellitus, experienced sudden‐onset blurry vision, diplopia, bilateral eyelid drooping, and loss of consciousness while working in the field. Upon examination at the tertiary care center, the patient displayed severe neurological impairment, including unconsciousness, nonreactive pupils, and lateral deviation of the eyes. These clinical features, along with the MRI findings of altered T2/FLAIR signal intensity lesions in bilateral thalami and the midbrain, strongly suggested bilateral thalamic infarction resulting from AOP occlusion.

The patient's clinical presentation in this case was characterized by sudden‐onset blurry vision, diplopia, bilateral ptosis, and loss of consciousness. The presence of lateral deviation in the bilateral eyes and nonreactive pupils pointed towards a significant midbrain involvement. The absence of neck rigidity was notable and helped distinguish this case from typical presentations of infectious causes, prompting further investigations into cerebrovascular etiologies. The clinical findings of nonreactive pupils and lateral eye deviation also align with midbrain involvement. The midbrain's role in regulating pupillary responses to light and eye movements is well‐documented. Nonreactive pupils and abnormal eye positioning are consistent with the broader neurological disruption associated with midbrain infarctions.

Sequelae of bilateral infarction in the paramedian thalamic region have been reported to include decreased levels of consciousness, alterations in mental status, apathy, memory loss, difficulty with language (aphasia), speech difficulties (dysarthria), motor deficits, and disinhibited behaviors.^6^, ^7^, ^8^ If the entire midbrain is affected, this leads to mesencephalothalamic or thalamopeduncular syndrome, which can include other oculomotor disorders and the involvement of long motor pathways.^9^, ^10^, ^11^, ^12^

The diagnosis of AOP occlusion relies on cerebral imaging studies, primarily MRI, since CT findings may appear normal.^13^ To identify AOP infarct early on, FLAIR and diffusion‐weighted imaging are the preferred imaging techniques.^4^, ^8^ Although an initial MRI brain scan may be normal, it cannot exclude AOP ischemic infarcts.^14^ Hence, it is advisable to consider a repeat MRI focused on the vertebrobasilar territory.^15^ While CT may be more accessible initially, MRI is essential and should be performed either as the primary or secondary imaging study. Ischemic lesions of the medial parts of both thalami, with or without rostral mesencephalic involvement, are observed.^4^ The midbrain ‘V' sign, which appears as a high‐intensity signal on axial FLAIR and diffusion‐weighted images along the pial surface of the midbrain in the interpeduncular fossa, has been reported in 67% of patients with AOP occlusion.^4^ In our case the MRI of the brain revealed altered T2/FLAIR signal intensity lesions in bilateral thalamus, including the midbrain, with restricted diffusion and blooming artifact. The non‐visualization of the right P1 segment and its origin from the right Pcom raised suspicion of bilateral upper midbrain infarction involving the AOP.

The main differential diagnosis involves bilateral thalamic venous infarction that complicates internal cerebral vein thrombosis.^13^, ^16^, ^17^ Although bilateral thalamic infarction can be part of the top of the basilar syndrome,^18^ it is uncommon to find isolated bilateral paramedian thalamic infarction.^18^ In our case, the working diagnosis was stroke; however, other less probable conditions could also lead to bilateral thalamic lesions. These conditions encompass thiamine deficiency,^19^ cerebral lupus,^20^ abscesses caused by fungi or toxoplasmosis,^21^ cerebral syphilitic gumma,^22^ and tumors.^23^ Additionally, in children, bilateral thalamic necrosis has been associated with influenza.^24^

The thalamic infarction can be linked to venous sinus thrombosis, resulting from occlusion of internal cerebral veins.^25^ Symptoms include headache, vomiting, and papilledema, leading to infarction with seizures, focal neurological deficits, and aphasia.^25^ Wernicke's encephalopathy is also included in the differential diagnosis because it results in lesions in bilateral thalami, periaqueductal gray tectal plate, dorsal medulla, and mammillary bodies.^26^ Infections and osmotic myelinolysis may be included in the differential diagnosis, as well.^25^

AOP infarcts should be considered as a possible diagnosis when dealing with altered mental status in elderly patients. According to a study, memory impairment was observed in 58% of patients, while coma was present in 42%, confusion in 53%, and vertical gaze palsy in 65% of patients.^5^

Thalamic dementia results from bilateral thalamic infarcts, not unilateral ones. It leads to memory impairment, confusion, and coma due to disconnection between the thalamus and cortex caused by a paramedian thalamic infarct.^1^

The standard treatment for an acute ischemic stroke depends on factors such as timing, lesion location, and contraindications to thrombolytics. For cases involving proximal cerebral artery occlusion, the recommended approach is the administration of recombinant tPA within 4.5 h of onset, combined with mechanical thrombectomy within 6 h.^27^ In contrast, AOP represents a distal artery of the posterior circulation with a small diameter, and information on the efficacy of mechanical removal is challenging to find. Due to its size, endovascular revascularization is rarely considered as an option for AOP infarcts, as it is not easily visualized.^28^

## 4 CONCLUSION

In conclusion, this rare case of bilateral thalamic infarction resulting from AOP occlusion emphasizes the intricate relationship between vascular anatomy, midbrain function, and clinical manifestations. The patient's abrupt onset of visual disturbances, diplopia, and eyelid drooping underscored the involvement of critical midbrain structures, notably the third cranial nerve. Advanced imaging techniques played a crucial role in diagnosis, revealing characteristic MRI findings. As such, this case serves as a reminder of the complexity of cerebrovascular events and the necessity for vigilant clinical assessment, leading to accurate diagnosis, and tailored management strategies.

## AUTHOR CONTRIBUTIONS

**Eliz Achhami:** Conceptualization; data curation; formal analysis; funding acquisition; investigation; methodology; project administration; resources; supervision; validation; visualization; writing – original draft; writing – review and editing. **Seshkanta Lamichhane:** Conceptualization; data curation; formal analysis; investigation; project administration; supervision; visualization; writing – original draft; writing – review and editing. **Satyam Mahaju:** Conceptualization; data curation; formal analysis; funding acquisition; investigation; methodology; resources; supervision; validation; visualization; writing – original draft; writing – review and editing. **Lukash Adhikari:** Conceptualization; data curation; formal analysis; funding acquisition; investigation; resources; validation; writing – original draft; writing – review and editing. **Ashim Kandel:** Formal analysis; funding acquisition; resources; writing – review and editing. **Anubhav Poudel:** Funding acquisition; investigation; project administration; resources; writing – review and editing.

## FUNDING STATEMENT

No funding was generated for this case report.

## CONFLICT OF INTEREST STATEMENT

The authors would like to declare that they have no competing interests.

## ETHICS STATEMENT

As case reports are exempt from ethical approval in our institution, our article which describes a case report does not require additional permissions from the Ethics committee.

## CONSENT STATEMENT

Full written informed consent was obtained from the patient for publication of her case, clinical images, and radiographic images. A copy of written consent can be made available to the editor in chief of this journal upon request.

## Data Availability

All the data generated or analyzed during this study are included in the manuscript.

## References

[1] Percheron G . The anatomy of the arterial supply of the human thalamus and its use for the interpretation of the thalamic vascular pathology. Z Neurol. 1973;205(1):1‐13. doi:10.1007/BF00315956 4126735

[2] Lasjaunias P , Berenstein ABK . Surgical Neuroangiography. Vol 1. 2nd ed. Berlin Springer‐Verlag; 2000:526‐562.

[3] EPOS™‐C‐0993. Accessed August 3, 2023. https://epos.myesr.org/poster/esr/ecr2015/C‐0993

[4] Lazzaro NA , Wright B , Castillo M , et al. Artery of percheron infarction: imaging patterns and clinical spectrum. AJNR Am J Neuroradiol. 2010;31(7):1283‐1289. doi:10.3174/AJNR.A2044 20299438 PMC7965474

[5] Lamot U , Ribaric I , Popovic KS . Artery of Percheron infarction: review of literature with a case report. Radiol Oncol. 2015;49(2):141‐146. doi:10.2478/RAON-2014-0037 26029025 PMC4387990

[6] Carrera E , Bogousslavsky J . The thalamus and behavior: effects of anatomically distinct strokes. Neurology. 2006;66(12):1817‐1823. doi:10.1212/01.WNL.0000219679.95223.4C 16801643

[7] Khanni JL , Casale JA , Koek AY , Espinosa del Pozo PH , Espinosa PS . Artery of Percheron infarct: an acute diagnostic challenge with a Spectrum of clinical presentations. Cureus. 2018;10(9):e3276. doi:10.7759/CUREUS.3276 30443447 PMC6235647

[8] Matheus MG , Castillo M . Imaging of acute bilateral Paramedian thalamic and mesencephalic infarcts. AJNR Am J Neuroradiol. 2003;24(10):2005‐2008. 14625223 PMC8148919

[9] Raut TP , Baheti G , Hinduja A , Makhija P , Ansari K , Sharma V . Sudden onset altered sensorium: artery of Percheron infarct. J Neurol Neurosci. 2017;8(4):212. doi:10.21767/2171-6625.1000212

[10] Reilly M , Connolly S , Stack J , Martin EA , Hutchinson M . Bilateral Paramedian thalamic infarction: a distinct but poorly recognized stroke syndrome. QJM an Int J Med. 1992;82(1):63‐70. doi:10.1093/OXFORDJOURNALS.QJMED.A068650 1438669

[11] Rangel‐Castilla L , Gasco J , Thompson B , Salinas P . Bilateral paramedian thalamic and mesencephalic infarcts after basilar tip aneurysm coiling: role of the artery of Percheron. Neurocirugia (Astur). 2009;20(3):288‐293. doi:10.1016/S1130-1473(09)70171-X 19575135

[12] Roitberg BZ , Tuccar E , Alp MS . Bilateral paramedian thalamic infarct in the presence of an unpaired thalamic perforating artery. Acta Neurochir. 2002;144(3):301‐304. doi:10.1007/S007010200040 11956945

[13] Lamboley JL , Le Moigne F , Have L , et al. Artery of Percheron occlusion: value of MRI. A review of six cases. J Radiol. 2011;92(12):1113‐1121. doi:10.1016/J.JRADIO.2011.08.007 22153043

[14] Cassourret G , Prunet B , Sbardella F , Bordes J , Maurin O , Boret H . Ischemic stroke of the artery of Percheron with normal initial MRI: a case report. Case Rep Med. 2010;2010:1‐4. doi:10.1155/2010/425734 PMC2838368 20300550

[15] Amin OSM , Shwani SS , Zangana HM , Muhammad E , Hussein H , Ameen NA . Bilateral infarction of paramedian thalami: a report of two cases of artery of Percheron occlusion and review of the literature. BMJ Case Rep. 2011;2011:bcr0920103304. doi:10.1136/BCR.09.2010.3304 PMC3062066 22715252

[16] de la Cruz‐Cosme C , Márquez‐Martínez M , Aguilar‐Cuevas R , Romero‐Acebal M , Valdivielso‐Felices P . Percheron artery syndrome: variability in presentation and differential diagnosis. Rev Neurol. 2011;53(4):193‐200. doi:10.33588/rn.5304.2011084 21780071

[17] Smith R , Hourihan MD . Investigating suspected cerebral venous thrombosis. BMJ. 2007;334(7597):794‐795. doi:10.1136/BMJ.39154.636968.47 17431266 PMC1852020

[18] Usón‐Martín M , Gracia‐Naya M . Top of the basilar artery syndrome: clinico‐radiological aspects of 25 patients. Rev Neurol. 1999;28(7):698‐701. doi:10.33588/rn.2807.98143 10363297

[19] Vortmeyer AO , Colmant HJ . Differentiation between brain lesions in experimental thiamine deficiency. Virchows Arch A Pathol Anat Histopathol. 1988;414(1):61‐67. doi:10.1007/BF00749739 3144804

[20] Vern BA , Butler M . Transient thalamic hypodensity in lupus erythematosus with generalized seizures. Neurology. 1983;33(8):1081‐1083. doi:10.1212/WNL.33.8.1081 6683809

[21] Gonzales GR , Herskovitz S , Rosenblum M , et al. Central pain from cerebral abscess: thalamic syndrome in AIDS patients with toxoplasmosis. Neurology. 1992;42(5):1107‐1109. doi:10.1212/WNL.42.5.1107 1579236

[22] Standaert DG , Galetta SL , Atlas SW . Meningovascular syphilis with a gumma of the midbrain. J Neuro‐Ophthalmol. 1991;11(3):139‐143. https://journals.lww.com/jneuro‐ophthalmology/Fulltext/1991/09000/Meningovascular_Syphilis_with_a_Gumma_of_the.2.aspx 1836794

[23] Hirano H , Yokoyama S , Nakayama M , Nagata S , Kuratsu J . Bilateral thalamic glioma: case report. Neuroradiology. 2000;42(10):732‐734. doi:10.1007/S002340000380 11110074

[24] Shinjoh M , Bamba M , Jozaki K , Takahashi E , Koinuma G , Sugaya N . Influenza A‐associated encephalopathy with bilateral thalamic necrosis in Japan. Clin Infect Dis. 2000;31(2):611‐613. doi:10.1086/313978 10987732

[25] Ferro JM , Canhão P , Aguiar de Sousa D . Cerebral venous thrombosis. Presse Med. 2016;45(12 Pt 2):e429‐e450. doi:10.1016/J.LPM.2016.10.007 27816347

[26] Kaya AH , Dagcinar A , Ulu MO , et al. The perforating branches of the P1 segment of the posterior cerebral artery. J Clin Neurosci. 2010;17(1):80‐84. doi:10.1016/J.JOCN.2009.03.046 20006506

[27] Rabinstein AA . Treatment of acute ischemic stroke. Continuum (Minneap Minn). 2017;23(1):62‐81. doi:10.1212/CON.0000000000000420 28157744

[28] 2018 AHA/ASA Stroke Early Management Guidelines–American College of Cardiology. Accessed July 30, 2023. https://www.acc.org/Latest‐in‐Cardiology/ten‐points‐to‐remember/2018/01/29/12/45/2018‐Guidelines‐for‐the‐Early‐Management‐of‐Stroke

